# Discovery of a new highly pathogenic toxin involved in insect sepsis

**DOI:** 10.1128/spectrum.01422-23

**Published:** 2023-10-03

**Authors:** Yuan Zhang, Hao Li, Fang Wang, Chang Liu, Gadi V. P. Reddy, Hu Li, Zhihong Li, Yucheng Sun, Zihua Zhao

**Affiliations:** 1 MARA Key Laboratory of Surveillance and Management for Plant Quarantine Pests, College of Plant Protection, China Agricultural University, Beijing, China; 2 Institute of Plant Protection, Ningxia Academy of Agricultural and Forestry Sciences, Yinchuan, China; 3 Department of Entomology, Lousiana State University, Baton Rouge, Los Angeles, USA; 4 Sanya Institute of China Agricultural University, China Agricultural University, Sanya, China; 5 Institute of Zoology, Chinese Academy of Sciences, Beijing, China; University of Edinburgh, Midlothian, United Kingdom

**Keywords:** *Steinernema*, *Xenorhabdus*, phenotypic variation, apoptosis, Kyn pathway, actinomycin D

## Abstract

Insect sepsis is a severe consequence that arises from the invasion of the hemocoel by symbionts of entomopathogenic nematodes and bacteria. In the present study, we unveiled the heightened virulence of the entomopathogenic nematode *Steinernema feltiae* and the entomopathogenic bacteria *Xenorhabdus bovienii*, which operate symbiotically, against the wax moth *Galleria mellonella*. Maximum mortality was observed at 25°C while the optimal infestation efficiency was 20 nematodes per host. After infestation, *G. mellonella* displayed rapid darkening and softening, accompanied by an escalated esterase activity at 9 h. The *X. bovienii*, released by *S. feltiae*, underwent substantial proliferation and discharged toxins that attacked hemocytes, thus triggering extensive hemolysis and sepsis. The host *G. mellonella* was usually killed within 24 h due to disseminated septicemia. Additionally, *X. bovienii* infestation led to the upregulation of metabolites like 3-hydroxyanthranilic acid. Strikingly, we identified the perilous actinomycin D, generated through kynurenine metabolites, representing a novel biomarker of insect sepsis. Furthermore, a comprehensive transcriptomic analysis unveiled a noteworthy upregulation of gene expression associated with actinomycin D. Overall, *X. bovienii* induced apoptosis and sepsis through actinomycin D production, indicating its pivotal role in infestation activity. These findings open up new avenues for studying the mechanism of sepsis and developing innovative biotic pesticides.

**IMPORTANCE:**

As a current biocontrol resource, entomopathogenic nematodes and their symbiotic bacterium can produce many toxin factors to trigger insect sepsis, having the potential to promote sustainable pest management. In this study, we found *Steinernema feltiae* and *Xenorhabdus bovienii* were highly virulent against the insects. After infective juvenile injection, *Galleria mellonella* quickly turned black and softened with increasing esterase activity. Simultaneously, *X. bovienii* attacked hemocytes and released toxic components, resulting in extensive hemolysis and sepsis. Then, we applied high-resolution mass spectrometry-based metabolomics and found multiple substances were upregulated in the host hemolymph. We found extremely hazardous actinomycin D produced via 3-hydroxyanthranilic acid metabolites. Moreover, a combined transcriptomic analysis revealed that gene expression of proteins associated with actinomycin D was upregulated. Our research revealed actinomycin D might be responsible for the infestation activity of *X. bovienii*, indicating a new direction for exploring the sepsis mechanism and developing novel biotic pesticides.

## INTRODUCTION

The wide application of chemical pesticides imposes a considerable burden on agricultural production, environmental sustainability, and ecosystem functions (1, 2). As a successful biocontrol strategy, the use of entomopathogenic nematodes (EPNs) has the potential to promote green pest management for the sustainable development of agriculture (3). EPNs are highly pathogenic toward insects, species specific, and environmentally friendly, thereby benefitting humans (4). The infective juveniles (IJs) of *Steinernema* spp. are the only free-living nematodes that enter and invade insect hosts via surface penetration (5, 6). Then, the EPNs release entomopathogenic bacteria (EPBs), tightly and longitudinally arranged in foregut vesicles, into the insect hemolymph (7). EPBs proliferate and generate a wide range of toxin molecules that kill the host within 48 h (8). The symbiosis of EPNs/EPBs induces physiological and biochemical responses in the host hemolymph (9). For example, esterases in the hemolymph of insects, which are a class of critical proteins that can affect the transport and metabolism of normal lipids and nerve conduction (10, 11), are upregulated for breakdown of the cytomembrane and disruption of the immune system (11). Then, overexpression of esterases leads to dysfunction or direct destruction in host hemocytes (12). The EPB *Xenorhabdus bovienii* can function alone to kill insect hosts, although both *Steinernema feltiae* and *X. bovienii* are involved in the lethality of insects (13, 14). To date, all *Xenorhabdus* species have exhibited phenotypic variation; they are usually isolated directly from EPNs in the primary form but some cells progress to the secondary form under nutrient and oxygen depletion during *in vitro* cultivation (15, 16). The primary forms of EPBs can attach to the surface of specific tissues in the hemocoel via flagella, producing a variety of immunosuppressive factors, toxin proteins, and specialized metabolites during infection that allow the host to die rapidly (17 – 20). However, the secondary forms of EPBs exhibit weaker motility and pathogenicity, but they have greater capacity for survival and reproduction, which allows them to better adapt to the *in vitro* environment (7, 13, 21, 22).

The *Xenorhabdus* bacterial genome encodes a vast variety of protein toxins and natural products (23). They generate a broad spectrum of insecticidal proteins, including Xpts (*Xenorhabdus* protein toxins), Txp40 (40-kDa toxin from *Xenorhabdus* and *Photorhabdus*), XhlA (cell surface-associated hemolysin), and Xax (24 – 26). In addition to these toxin proteins, natural products can kill host insects and prevent cadaver infection by inhibiting the growth of bacteria, fungi, and protozoa during infestation (27 – 29). Several natural products encoded by polyketide synthetases (PKSs) and nonribosomal peptide synthetases (NRPSs), with a wide range of biological activities, have been isolated from different *Xenorhabdus* strains, including xenoamicins (30), lipodepsipeptides (31), xenematides (32), szentiamide (33), xenobactin (34), xenocoumacins (35), fabclavines (36), pristinamycin (37), xenortides (38), rhabdopeptides (39), bicornitun (40), PAX peptides (41), cabanillasin (42), and nemaucin (42). In addition, bacteriocins encoded by ribosomes, indole and its derivatives, amino acid derivatives, and other specialized metabolites are produced, such as xenorhabdicin (43), xenocin (44), nematophin (45), rhabduscin (46), benzylideneacetone (47), furanones (48), dithiopyrroles (49), tryptamide (50), and a few unnamed peptides (51, 52). In recent years, it has been found that microorganisms, especially those that produce the lethal antibiotics actinomycins, can utilize L-tryptophan (Trp), an essential primary metabolite, for catabolic and anabolic processes (53). Actinomycins are a large class of orange-red chromophore compounds harboring a cyclic pentapeptide lactone linked by two amide bonds (54). Our previous study showed that the Trp metabolite 3-hydroxyanthranilic acid (3-HAA) was somewhat toxic to *Galleria mellonella* and might play a potential role in mediating the killing of prey by EPNs but the natural products responsible for the differences in the virulence of the two forms of the EPB are unclear (55).

We explored three research topics in this study. First, we studied the environment-dependent infestation by EPNs and their phenotypes associated with insect mortality. Second, we performed multiomics (e.g., metabolomics and transcriptomics) analysis of the virulence of the primary and secondary forms of EPBs, as well as examination of the synthetic pathways of potential biomarkers. Third, we addressed the phenotypic differences in EPBs and infestation mechanisms. Metabolomics was used to reveal the virulence effects of potential toxins via synthetic pathway analysis. We hope that these findings will be helpful in facilitating the study of the pathogenic mechanisms of EPNs.

## RESULTS

### Life history of *S. feltiae*

**Fig 1:**
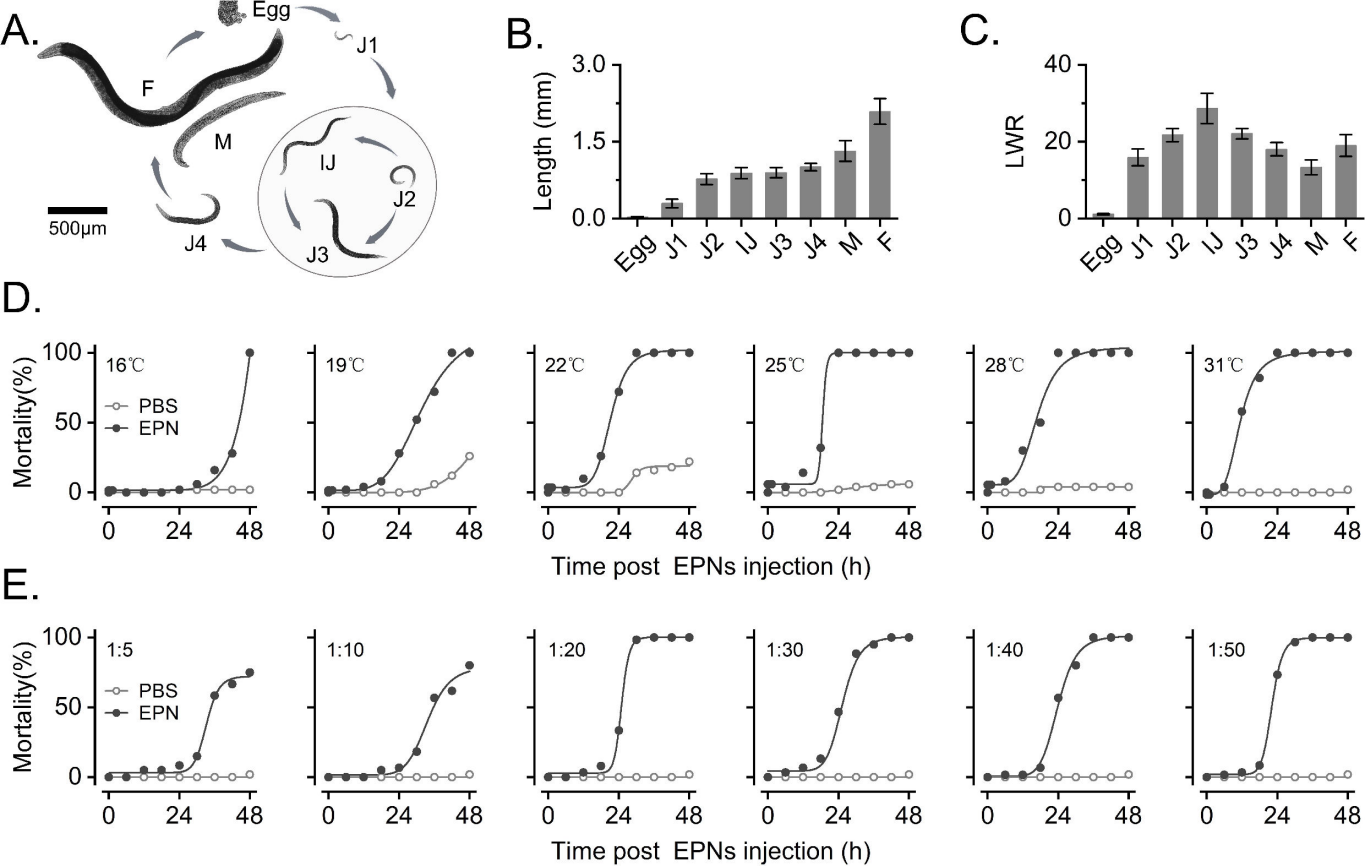
Life history of and infestation by of the symbionts *S. feltiae* and *X. bovienii*. (**A**) Morphometrics of *S. feltiae* in different stages. (**B**) Body length of *S. feltiae*. (**C**) LWR (length-to-width ratio) of *S. feltiae*. (**D**) Temperature-dependent effects of *S. feltiae* infestation on the mortality of *G. mellonella*. (**E**) Effect of *S. feltiae* infestation on the mortality of *G. mellonella* at different doses.

The long lifespan of EPNs includes the egg stage, four larval stages, and the adult stage within the ~13 days they spend in their insect hosts (Fig. 1A). A single female adult EPN can produce approximately 800 eggs, and an individual *G. mellonella* can release 41,056.67 ± 12,699.65 IJs into the surrounding environment (Fig. 1A). The body lengths of male and female EPNs were 1.32 ± 0.20 and 2.09 ± 0.25 mm, respectively (Fig. 1B). Additionally, the body length of IJs was nearly 30 times the greatest body width and the length was the most distinct feature of the IJs compared with the nematodes at other stages (Fig. 1C).

### Insecticidal activity of *S. feltiae*

The increasing temperature could enhance the mortality of *G. mellonella*, which indicates that it takes 24 h to achieve 100% mortality post IJ injection at 25°C (Fig. 1D). The mortality of *Bactrocera dorsalis* similarly peaked at 71.9% at 25°C (Fig. S1A). Therefore, the optimal temperature for *S. feltiae* and *X. bovienii* infestation of insect prey was 25°C, with high insecticidal activity. The EPN/EPB symbiotic infestation of *G. mellonella* was significantly higher than that of *B. dorsalis* (*F* = 16.00, *P* < 0.001). When the same dose was instilled onto the body surface of *B. dorsalis*, virulence attenuation and hysteresis were observed (Fig. 1E; S1B). The optimum density of IJs was assayed in the range of 20–50 IJs/larva, which showed significantly higher mortality at 48 h compared with 5–10 IJs/larva (*F* = 17.07, *P* < 0.001). These observations indicate that both temperature and IJ dosage could affect the infestation efficacy, which means an optimal infestation condition (20 IJs/larva at 25°C).

### Esterase and hemocyte characteristics of infected *G. mellonella*

**Fig 2:**
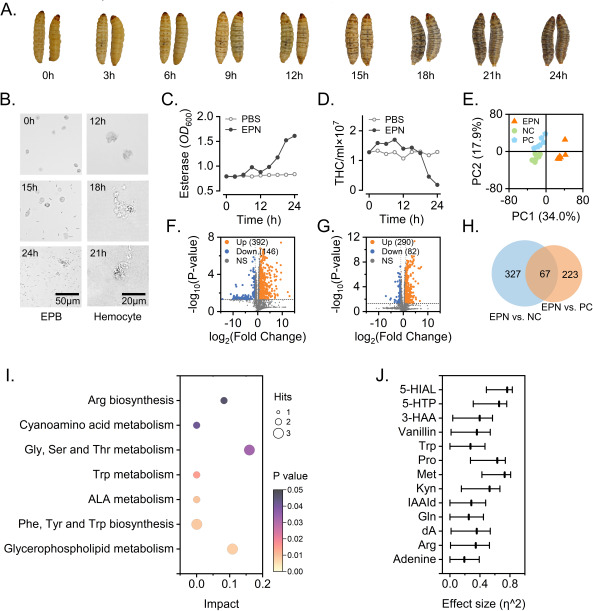
Pathologic phenotype and metabonomic analysis of the hemolymph. (**A**) Infestation processes of the EPN/EPB symbionts (*S. feltiae* on *G. mellonella*). (**B**) Hemocoel response of *G. mellonella* to EPN/EPB infestation. (**C**) Esterase activity of *G. mellonella*. (**D**) Changes in the THC after injection of *S. feltiae*. (**E**) PCA score plot for *S. feltiae* injection (EPN), untreated samples used as negative control (NC), and sterile water injected as a positive control (PC). (**F**) Volcano plots (EPN vs. NC). The horizontal coordinate indicates the change in gene expression [log_2_ (fold change)], and the vertical coordinate indicates the significance level [−log_10_ (*P* value)]. Red and green dots represent the upregulated genes and downregulated genes, respectively. (**G**) Volcano plots (EPN vs. NC). (**H**) Venn diagram analysis of significantly differentially abundant compounds in EPN vs. NC and EPN vs. PC. (**I**) Results of pathway analysis. (**J**) Results of effect size analysis.

The infected *G. mellonella* turned gray or black, softened 15 h postinjection, and died within 24–48 h (Fig. 2A). Intriguingly, the infested *G. mellonella* had higher esterase activity in the hemolymph and peaked at 9 h postinjection (Fig. 2C). The hemocytes began to exhibit cell membrane lysis. A few *X. bovienii* were found in the hemolymph 12 h after IJ infection (Fig. 2B). In contrast, the number of hemocytes exhibited a slight increase at 9 h and then rapidly decreased, with a large number of lysed hemocytes present in the hemolymph (Fig. 2D).

### Phenotypic variation characteristics and virulence of *X. bovienii*

**Fig 3:**
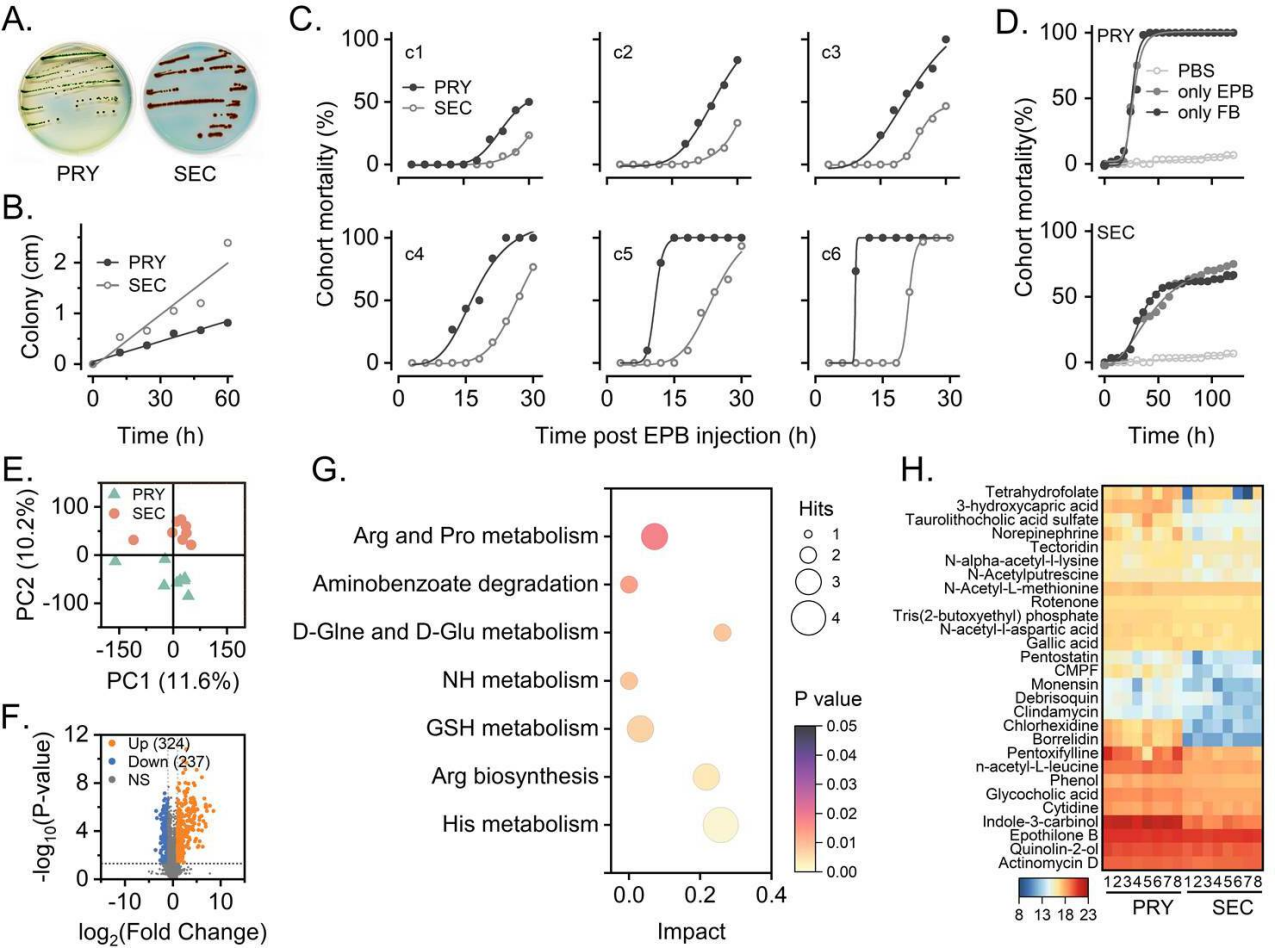
Pathologic phenotype and metabolomic analysis of *X. bovienii*. (**A**) The colony phenotype of the primary (PRY) and secondary (SEC) forms of *X. bovienii* on NBTA. (**B**) *X. bovienii* survival capability analysis in vitro. (**C**) The effects of *X. bovienii* for the primary and secondary forms on infestation and the mortality of *G. mellonella*. (**D**) Effect of the two forms of *X. bovienii*, EPB cells alone and cell-free fermentation broth (FB) alone on pathogenic ability. (**E**) PCA score plot of the primary and secondary EPB fermentation broths. (**F**) Volcano plots of PRY vs. SEC. (**G**) Heatmap of differential compound expression in the fermentation broth of the primary and secondary forms. Differential expression is shown as log_2_ (fold change). (**H**) The enriched Kyoto Encyclopedia of Genes and Genomes (KEGG) pathways of the differential metabolites in PRY compared with SEC.

The primary form of *X. bovienii* causes the insect cadaver to become flaccid and liquefied, while the secondary form causes rigidity (Fig. 3A). The secondary form of *X. bovienii* grew more rapidly than the primary form (*F* = 3.128, *P* < 0.001). Thus, the primary form exhibited small green colonies on NBTA, while the secondary form grew as red colonies with larger diameters (Fig. 3B). The primary form had significantly higher toxicity toward insect hosts than the secondary form during infestation (*F* = 0.235, *P* < 0.05 Fig. 3C). At 120 h, both the EPB alone and cell-free fermentation broth showed the same toxic activity, both at 100%, toward the insect host for the primary form of *X. bovienii* with no difference observed; although the secondary form exhibited relatively weaker toxicity, there was no significant difference between the EPB alone and the cell-free fermentation broth (*F* = 1.839, *P*＞0.05) (Fig. 3D). Therefore, the active metabolites causing insect mortality were present in the cell-free fermentation broth of the primary form of *X. bovienii*.

### Screening and identification of insecticidal substances

A total of 158 differential metabolites (*P* < 0.05, fold change > 1.5, and CV < 20) were identified in the positive ion mode in the *G. mellonella* hemolymph metabolomics sample. These metabolites included organoheterocyclic compounds, phenylpropanoids, polyketides, benzenoids, organic acids and derivatives, organic oxygen compounds, organosulfur compounds, nucleosides, nucleotides, and their analogs. Significant differences were observed between the principal component analysis (PCA) scores of the IJ-injected group and those of the water-injected group and the untreated control group. In addition, the PCA plots revealed differences in metabolic substances among the three groups (Fig. 2E). In total, 392 chemicals were considerably upregulated in the treatment group relative to the negative control group, whereas 290 compounds were significantly upregulated in the treatment group close to the positive control group (Fig. 2F and G). Sixty-seven chemicals were discovered to be jointly upregulated (Fig. 2H). The pathway topology analysis revealed eight metabolic pathways: glycerophospholipid metabolism; phenylalanine (Phe), tyrosine (Tyr), and Trp biosynthesis; alpha-linolenic acid (ALA) metabolism; Trp metabolism; glycine (Gly), serine (Ser), and threonine (Thr) metabolism; cyanoamino acid metabolism; and arginine (Arg) biosynthesis (Fig. 2I). Through effect size analysis, 5-hydroxyindolealdehyde (5-HIAL), methionine (Met), and 5-hydroxytryptophan (5-HTP) were identified as the top ranked among all the potential chemicals.

By analyzing differences in fermentation broth between the primary and secondary forms of *X. bovienii*, 107 and 57 metabolites were obtained in the positive and negative ion modes, respectively. These metabolites included alkaloids and their derivatives; benzenoids; hydrocarbon derivatives; lignans, neolignans, and related compounds; lipids and lipid-like molecules; nucleosides, nucleotides, and their analogs; organic acids and their derivatives; organoheterocyclic compounds; and phenylpropanoids and polyketides. The PCA score plots showed that the primary and secondary forms were mainly separated along t2 (Fig. 3E). Volcano plots showed that 324 substances were significantly upregulated in the primary form of the EPB (Fig. 3F). Pathway analysis revealed that the substances with defined structures were mainly enriched in histidine (His) metabolism, Arg biosynthesis, glutathione (GSH) metabolism, nitrogen (NH) metabolism, D-glutamine (Gln) and D-glutamate (Glu) metabolism, aminobenzoate degradation, and Arg and proline (Pro) metabolism (Fig. 3H).

**Fig 4:**
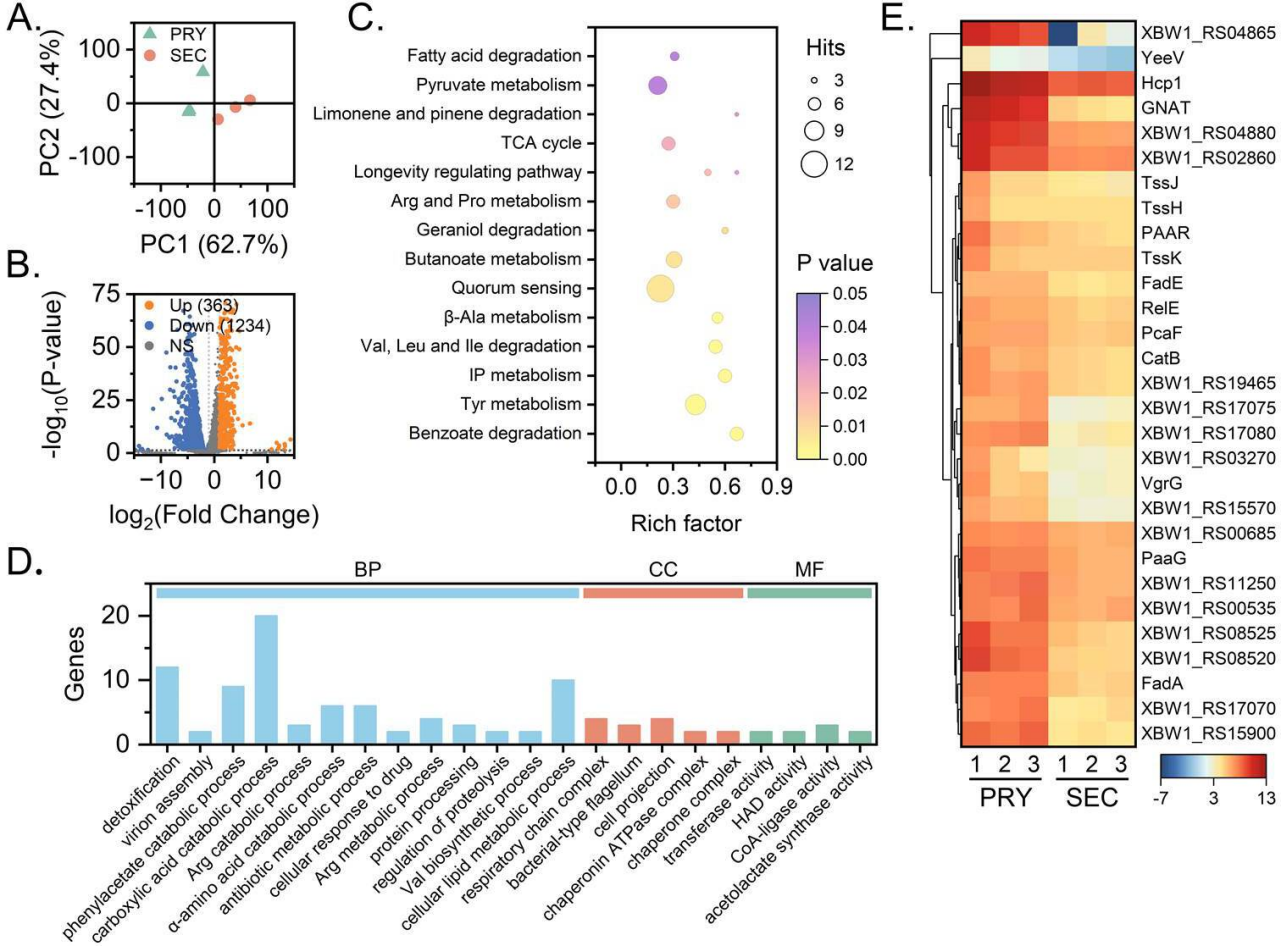
Transcriptomic analysis of the primary and secondary forms of *X. bovienii*. (**A**) PCA score plot of the primary (PRY) and secondary (SEC) forms of EPB. (**B**) Volcano plots. The horizontal coordinate indicates the change in gene expression (PRY vs. SEC) [log_2_ (fold change)], and the vertical coordinate indicates the significance level [−log_10_ (*P* value)]. The upregulated genes and downregulated genes are represented by red and green dots. (**C**) The enriched KEGG pathways of differentially expressed genes in the PRY form compared with the SEC form. (**D**) Gene function classification (GO). (**E**) Heatmap of the expression of the T6SS and toxin protein-associated genes in the PRY and SEC forms of the bacteria. Differential expression is shown as the log_2_ TPM of the PRY form of *X. bovienii*/TPM of the SEC form of *X. bovienii*.

The results of transcriptomic analyses showed that 4,523 genes were mapped to the reference genome, and the PCA score plots showed that the samples of the primary and secondary forms of the bacterium were well separated (Fig. 4A). The volcano plot showed that 1,597 genes were differentially expressed in the transcriptome, of which 363 were significantly upregulated and 1,234 were downregulated. The upregulated genes were enriched in metabolic pathways involving aromatic compounds; Phe metabolism; benzoate degradation; Tyr metabolism; inositol phosphate (IP) metabolism; valine (Val), leucine (Leu), and isoleucine (Ile) degradation; β-alanine (Ala) metabolism; and the tricarboxylic acid cycle (TCA) (Fig. 4B and C). Again, GO function enrichment showed that the significantly different genes had 95 annotations attributed to biological processes, 69 to cellular components, and 90 to molecular functions. Numerous activities linked to xenobiotic and toxin metabolism were enriched in the biological process category, while processes related to bacterial flagellum metabolism were the most abundant in the cellular component category. Additionally, many genes associated with type VI secretion system (T6SS) assembly, methyltransferases, and NRPSs were considerably upregulated; however, this pathway was not significantly enriched (Fig. 4D and E). Comparison of all the enriched pathways at the metabolomic and transcriptomic levels showed that the Arg and Pro metabolism pathway was present in both the bacterial transcriptome and the supernatant metabolome.

### Toxicity of potential insecticidal substances

**Fig 5:**
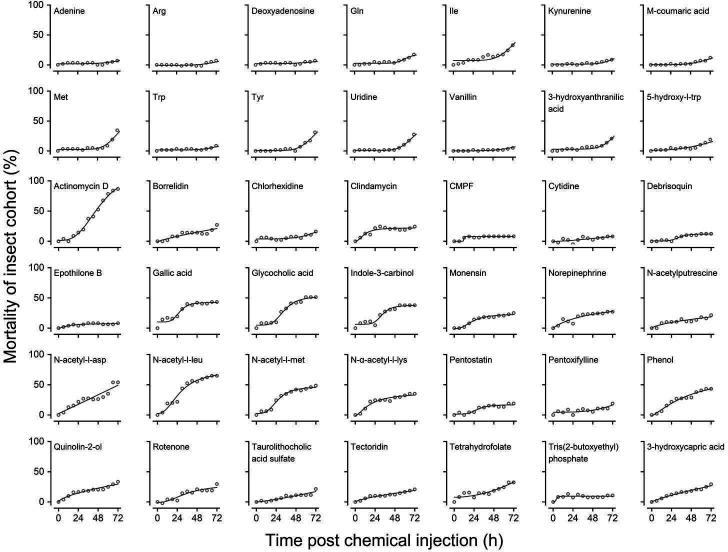
Toxicity of potential biomarkers in *G. mellonella*. In total, 40 substances from metabolome analysis were tested in the survival analysis.

In total, 40 components, including amino acids and their derivatives, antibiotics, and macrolides, were finally screened for toxicity verification. Actinomycin D showed high insecticidal activity (*F* = 0.49, *P* < 0.001). Additionally, N-acetyl-L-Leu (*F* = 3.23, *P* < 0.001), N-acetyl-L-aspartic acid (Asp) (*F* = 8.91, *P* < 0.01), and glycocholic acid (*F* = 1.13, *P* < 0.001) had high hemocoelic toxicity toward the insect host *G. mellonella*. Actinomycin D exhibited the highest hematological toxicity, with a cumulative mortality rate of 86.49% at 72 h (Fig. 5). The mortality trend in *B. dorsalis* was the same as that of *G. mellonella* for each chemical, with actinomycin D (*F* = 5.67, *P* < 0.05) lethality ranked at the top again (Fig. S2). Interestingly, N-terminally acetylated amino acids, such as N-acetyl-L-Asp, N-acetyl-L-Met, and N-acetyl-L-Leu, showed higher insecticidal activity regardless of the method of exposure or injection.

### Regulation of sepsis in the hemolymph by actinomycin D

**Fig 6:**
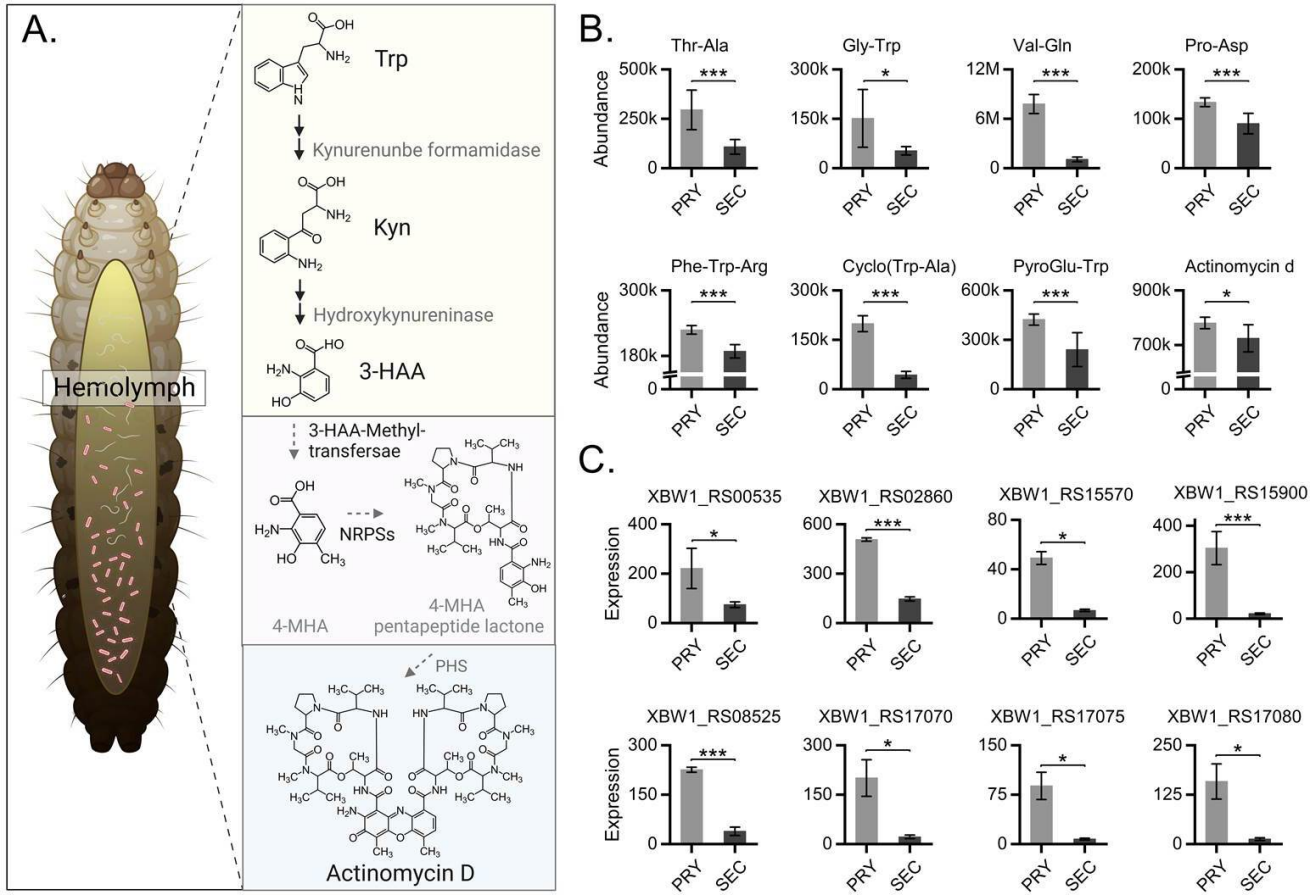
Biosynthesis of actinomycin D in the primary form of *X. bovienii*. (**A**) Biosynthetic pathway for actinomycin D involved in sepsis caused by EPB invading *G. mellonella*. (**B**) The abundance of related substances in fermentation broth metabolomics. (**C**) Expression of methyltransferase- and NRPS-related genes in the two forms of bacteria. *P < 0.05, ***P < 0.01.

4-Methyl-3-hydroxyanthranilic acid (4-MHA), containing an *o*-aminophenol group, was upregulated in kynurenine (Kyn) metabolism in the Trp pathway; 4-MHA and the five amino acids with the peptide lactone rings are subsequently assembled into the 4-MHA pentapeptide lactone. Two 4-MHA pentapeptide lactones undergo dimerization to produce the phenoxazinone actinomycin chromophore (Fig. 6A). Multiple amino acids with pentapeptide lactones in the form of dipeptides were upregulated in the hemolymph of infected *G. mellonella* (Fig. 6B). Some genes related to the methyltransferases responsible for converting 3-HAA to 4-MHA and NRPSs were found to be upregulated in the primary form of *X. bovienii*. XBW1_RS00535, XBW1_RS02860, XBW1_RS15570, and XBW1_RS15900 encode methyltransferases, and the rest are NRPS-associated genes (Fig. 6C).

## DISCUSSION

Although it has been known for many decades that infestation with EPNs/EPBs leads to efficient insect sepsis, little is known about the mechanism and biomarkers produced by them (56). Our ﬁndings were consistent with previous results showing that EPBs were indeed capable of causing sepsis in insects and were highly virulent. Meanwhile, EPB invasion stimulates the immune response of host insects, such as by enhancing esterase activity and increasing the abundance of immune cells (11, 57). We found that the change in esterase activity disrupted the balance of the insect immune system and the resulting fat body impairment prevented further resistance to dangerous EPB invasion (55). As in previous studies, the insect hemolymph infected by IJs showed a blurred nuclear boundary, then the hemocyte membrane was utterly broken, and the contents of the cells flowed out; eventually, the cells became apoptotic (58). It was speculated that the increase in the THC in the early stage might have been due to the rapid division of protohemocytes and the pinocytosis and phagocytosis of bacteria. Over time, the EPB aggravated the destruction of the host defense system and began to multiply in large numbers, causing hemocyte deformation, transformation, and disintegration. Finally, the number of hemocytes was reduced to a minimum. Interestingly, there were differences in the insecticidal activity of the two forms of the EPB. They also differed markedly in phenotype compared with that in which IJ injection led to insect sepsis (7). Other studies have shown that only when the EPB carried by the EPN was the primary form were the carcasses swollen and “floppy.” In contrast, the body a secondary form for killing maintained a rigid exoskeleton (59). This indicates that the EPN at the IJ stage specifically expresses some products with activity toward insect tissues and the expression of these products might be triggered by only the primary form of the EPB (60). Furthermore, there was also a significant difference in the toxicity of the cell-free fermentation broth of the two forms of the EPB and the insecticidal activity of the secondary form was relatively weak. In conclusion, our results demonstrated that the symbiotic EPB in IJs was a highly toxic primary form and key to the septicemia and insecticidal activity of IJs. The critical biomarker was also present in its cell-free fermentation broth.

Recent studies have shown that bacterial sepsis might be associated with immune cell apoptosis; it plays a critical role in biological development, self-protection, and defense against interference from external factors (61). Many compounds have been found to initiate the apoptotic program of cells, such as actinomycins of the cytotoxic antibiotic drug family. Actinomycin D is the most common actinomycins, as it is an iso-actinomycin composed of similar amino acids, including Thr, D-Val, Pro, sarcosine (Sar), and N-methylvaline (MeVal), where the source of sarcosine is Gly (62). Actinomycin D was detected in our results and exhibited extremely high insecticidal activity. Paramanathan et al. indicated that actinomycin D can block DNA-dependent mRNA synthesis by binding to DNA, thereby impeding protein synthesis, inhibiting cell division, and causing apoptosis (63). An essential precursor for actinomycin D synthesis, 4-MHA, is a unique compound derived from Trp and occurs in only the actinomycete family of bacteria; more common than 4-MHA in nature is its homolog 3-HAA (64). It is speculated that Trp, as the essential amino acid in organisms, is largely consumed and used to synthesize a variety of substances that play a role in host insects. These findings prove that Trp metabolism, especially Kyn metabolism, plays a critical role in the interactions of pathogens and host insects and may have a regulatory effect on immune cells. Because the expression levels of related substances and enzymes of the Trp metabolic pathway in the two forms of *X. bovienii* were significantly different, which was probably also related to the synthesis and expression of actinomycin D, we concluded that the Trp pathway was the critical metabolic pathway influencing EPB toxicity and that actinomycin D was the biomarker responsible for the difference in insecticidal activity of the two forms of *X. bovienii*.

Moreover, other compounds in this study also showed some insecticidal activity. They may play a synergistic role in insect killing or be of great importance in other processes, such as bacteriostasis. These compounds included glycocholic acid, with fat-dissolving properties and high cytotoxicity, as well as some acetylated amino acids, and all of them exhibited some pathogenicity toward *G. mellonella* (65, 66). Previous studies have shown that N-acetyltryptamine does have higher nematocidal, antibacterial, and insecticidal activities than tryptamine due to the presence of N-acetyl groups (67). However, whether *X. bovienii* can use the above compounds to synthesize substances that have insecticidal activity in insect-nematode-bacteria interactions, such as actinomycin D, and actinomycin D plays a role in causing insect sepsis remain unclear. Therefore, in-depth investigation of the metabolites of the mechanisms of action of the aforementioned compounds, as well as the metabolites of the Kyn pathway, could contribute to the development of novel insecticide lead compounds.

Taken together, after the EPN/EPB was delivered into the hemolymph of the host insect, the *G. mellonella* body gradually exhibited graying with black speckles and then rapidly disintegrated, leading to death. The actinomycin D content in the fermentation broth of the primary form of *X. bovieniii* is higher compared to that in the secondary form, which may contribute to the higher pathogenicity of the primary form bacteria. Through the combined analysis of transcriptomics and metabolomics of both forms of *X. bovienii*, several precursor substances and enzyme activities involved in actinomycin D synthesis were found to be significantly upregulated in the primary form *X. bovienii*. Considering the biological activity of actinomycin D in inducing cell apoptosis and exhibiting toxicity to insects, it can be speculated that actinomycin D produced by *X. bovienii* may contribute to insect sepsis. However, the specific mechanisms by which actinomycin D causes insect sepsis remain unclear. Therefore, in-depth investigation of the mechanisms of action of actinomycin D in insect sepsis would be beneficial for understanding its important role in biological control.

### Conclusion

In this study, we discovered new biomarkers and revealed the EPN/EPB infection mechanism on insect hosts that intuitively interact using ecological observations and omics techniques. EPBs may cause host insect hemolymph septicemia by releasing a series of biomarkers (e.g., actinomycin D, glycocholic acid, and acetylated amino acids). By jointly analyzing the hemolymph metabolome, fermentation broth metabolome, and transcriptome, we found that actinomycin D might be the critical biomarker of *X. bovienii* and may cause hemolymph septicemia. In addition, the synthesis of actinomycin D in *X. bovienii* was also summarized based on the synthesis in *Streptomyces* spp. In conclusion, we found that both temperature and IJ dose significantly impacted the pathogenic capacity of EPNs and the host insects could undergo a large degree of sepsis both at very low IJ doses and in the cell-free fermentation broth of EPBs. We demonstrated that the primary form of *X. bovienii* is the key factor underlying host death and that these biomarkers with lethal activity were present in its fermentation broth. After EPB infection, the *G. mellonella* surface or the esterase and hemocytes in the body change dramatically, which is considered necessary for insect immunity. Therefore, these results will be helpful in exploring the biocontrol potential of EPNs/EPBs and biocontrol technology. This study may provide a new model for generating more effective, safe, and environmentally sustainable chemical insecticides to protect people and animals from the effects of hazardous synthetic insecticides.

## MATERIALS AND METHODS

### Experimental species

*S. feltiae* SF-SN, *G. mellonella,* and *B. dorsalis* were preserved and bred at the MOA Key Laboratory of Pest Monitoring and Green Management, China Agricultural University.

*X. bovienii* was isolated from its symbiont *S. feltiae* SF-SN. Permanent stocks of all cultures were stored in Luria-Bertani (LB) broth (10 g/L tryptone, 5 g/L yeast extract, and 5 g/L NaCl at pH 7.0) supplemented with 20% glycerol at −80°C. Furthermore, *X. bovienii* was purified at 25°C under dark conditions in LB broth. All *X. bovienii* isolates were subsequently inoculated (1:100, vol/vol) in fresh LB medium and incubated in a rotary shaker until the optical density (OD_600_) was 0.8 (mid-logarithmic phase). When appropriate, the medium was supplemented with antibiotics at different concentrations (ampicillin, 150 µg/mL; kanamycin, 50 µg/mL) (19).

The EPNs were propagated as previously described and collected using the White trap technique with slight modifications, and nematodes at different stages within cadavers of *G. mellonella* were observed (68). The *G. mellonella* larvae were placed in Petri dishes (90 × 15 mm) containing a double layer of filter paper soaked with IJ suspension at 25°C. The newly emerged IJs from the cadavers were collected with a White trap, washed with sterile water three times, and stored in 250-mL tissue culture flasks at 13–15°C.

*G. mellonella* (Lepidoptera: Pyralidae) larvae were reared on an artificial diet in the laboratory at 25°C and 70 ± 5% RH in constant darkness (69). Larvae of *B. dorsalis* (Diptera: Tephritidae) were reared on an artificial diet in an environmentally sound, circulating, intelligent, artificial climate chamber at 25°C, 70 ± 5% RH, and 14L:10D (70).

### EPN virulence assay

The virulence of IJs at different temperatures and densities was determined by a mortality assay. Both *B. dorsalis* and *G. mellonella* larvae were surface sterilized using 70% (vol/vol) ethanol, washed with sterile water, and anesthetized on ice for insect pretreatment. Nematode suspensions of 20 IJs/larva were utilized for the virulence assays at 16, 19, 21, 25, 28, and 31℃. EPN densities of 5 IJs/larva, 10 IJs/larva, 20 IJs/larva, 30 IJs/larva, 40 IJs/larva, and 50 IJs/larva were used for virulence assays at 25°C with phosphate-buffered saline (PBS) as a control. Approximately 10 µL of nematode suspension was added dropwise onto the body surface of *B. dorsalis* and injected into the body cavity of *G. mellonella* from the third pair of gastropodia using a sterilized microsyringe (Hamilton 1702 RN, 25 μL). All treated insects were placed individually into the wells of 12-well culture plates with a double layer of wet filter paper. Insect larvae treated with different densities of nematode suspension were placed separately in a circulating, intelligent, artificial climate chamber at 25°C, 70 ± 5% RH, and 14L:10D. Additionally, insect larvae treated with different temperatures were placed in the same consistent conditions. The insect larvae were considered dead from EPN infection when they did not respond to prodding with a needle tip and their bodies showed typical signs of darkening and softening. The cohort mortality of *G. mellonella* was checked every 6 h, and that of *B. dorsalis* was checked every 12 h. In total, 50 larvae of *G. mellonella* with five replicates and 110 larvae of *B. dorsalis* with 11 replicates were used to examine the mortality responses to EPN infestation.

### Hemocyte observation

To determine hemocyte pathology, observation was performed using a larva of *G. mellonella* injected with 20 IJs. The *G. mellonella* abdominal segment proleg was cut with curved tissue scissors, and the hemolymph was allowed to ooze out on a clean, oil-free glass microscope slide. The hemolymph was quickly diluted 20-fold with 1–2% glacial Turek solution and transferred to a disposable cell counting plate for counting under a microscope. The preparations were discarded if the cells aggregated or were distributed unevenly within the chamber (57). The changes in the pathological and total hemocyte count (THC) of hemocytes of the defined infected insects and control hemolymph were recorded every 3 h within 24 hr.

### Esterase activity assay

Fifty microliters of hemolymph was added to a mixed solution of PBS (0.45 mL, 0.04 mol/L) and α-naphthyl acetate solution (3 mL, 3 × 10^−4^ mol/L). Then, 0.5 mL of a mixed solution (2:5, vol/vol) of fast blue RR salt solution (10 g/L) and SDS (15 g/L) was added for approximately 30 min to determine the OD_600_ of the hemolymph culture to characterize the esterase activity. The OD_600_ of the defined infected insects and control hemolymph was recorded every 3 h within 24 hr.

### Bacterial viability and virulence assay

Stable primary and secondary forms of *X. bovienii* were purified by differential separation by color on NBTA selective medium (5 g/L beef peptone, 10 g/L tryptone, 5 g/L NaCl, 0.025 g/L bromthymol blue, 0.04 g/L triphenyl tetrazolium chloride, and 15 g/L agar) (71). The viability of the primary form and secondary form of *X. bovienii* on NBTA was characterized using colony diameter. A sterilized toothpick was dipped in the bacterial culture solution in the mid-logarithmic phase, and the toothpick was used to inoculate the center of the NBTA medium; the colony diameter was measured every 12 h for a total of 60 h.

Single colonies for the primary and secondary forms of *X. bovienii* from each NBTA selective medium were selected and grown overnight in 5 mL of LB broth supplemented with 0.1% (wt/vol) sodium pyruvate (LBP) to the mid-logarithmic phase. One milliliter of each bacterial culture was centrifuged at 12,000 rpm for 5 min to obtain cell-free fermentation broth and EPB cells. Bacterial cells were washed with PBS three times by centrifugation, and the sample was diluted five times with a 1:10 serial dilution to obtain a bacterial cell suspension of lower concentration. Ten microliters of inoculum was used for each form at the test concentration was injected into *G. mellonella* (72). PBS without fermentation broth and EPB cells was used as a control. The cohort mortality was checked every 6 h for 120 h. A total of 60 larvae, divided into six groups for each form at the test concentration, were used, and the other experimental settings were the same as those described above.

### Omics analyses

#### Hemolymph metabonomics

The hemolymph of *G. mellonella* was collected after injection of 20 IJs and transferred to a 1.5-mL centrifuge tube. The untreated hemolymph sample from *G. mellonella* was used as a negative control, and to eliminate any errors caused by injection methods such as piercing, sterile water injection was used as a positive control. In each test, *G. mellonella* was separated into six groups and 50 µL of hemolymph was collected from each group. Each sample was added to precooled MeOH-ACN (1:1, vol/vol), mixed well by shaking, and centrifuged at 12,000 rpm and 4℃ for 10 min. Then, the supernatant was vacuum dried and 40 µL of MeOH-H_2_O (1:1, vol/vol) was added for reconstitution. The fermentation broth sample was shaken for 30 min, centrifuged at 12,000 rpm and 4℃ for 10 min, filtered through a 0.1-µm membrane, and then used for analysis. A UPLC-HRMS system (UPLC, ACQUITY UPLC I-Clas Bio, Waters; MS, Q-Exactive Focus, Thermo Scientific) equipped with a heated electric spray ionization (HESI) source was used for hemolymph extraction, and MS analysis was performed in the positive ion mode. The analysis program setting was as previously described with slight modification (73).

### Cell-free fermentation broth metabolomics

The cell-free fermentation broth of *X. bovienii* was collected and stored on dry ice with eight replicates. The mixture was shaken slowly at 4°C, and an appropriate amount of fermentation broth was added to precooled MeOH-ACN-H_2_O (2:2:1, vol/vol), vortexed, ultrasonicated at 4°C for 30 min, and centrifuged at 14,000 rpm and 4°C for 20 min. The fermentation broth was dried *in vacuo*, reconstituted in 100 µL of ACN-H_2_O (1:1, vol/vol), vortexed, and centrifuged at 14,000 rpm at 4°C for 15 min. Fermentation broth samples were retained for metabolite profiling using an ultra-high performance liquid chromatograph (UHPLC ) (1290 Infinity LC, Agilent Technologies) coupled to a time-of-flight (TOF) quadrupole platform (AB Sciex Triple-TOF 6600) in positive and negative ion modes. Xcalibur 4.0 software (Thermo Fisher) was used for data acquisition and to calculate peak areas, and SIEVE 2.1 software (Thermal Scientific) was used for optimization of peak alignment and component extraction from the original data. Metabonomics databases (HMDB, KEGG, LIPID, MAPS, MassBank, MeSH, METLIN, and PubChem) were used for metabolite identification, and then, the fragment ion spectrum was matched with the candidate compound by MS/MS spectral database matching. Finally, MetaboAnalyst 3.0 was used to screen and analyze the correlations and pathways of differential metabolites.

### Transcriptome sequencing

The difference in virulence between primary and secondary forms of *X. bovienii* was represented by the differential expression of genes measured by transcriptomic analysis. The fermentation broth in the mid-logarithmic phase was centrifuged to collect bacterial cells for transcriptomic sequencing on the Illumina HiSeq platform (Illumina, Inc., San Diego, CA, USA). The sequencing results were assembled using Rockhopper according to the reference genome (*X. bovienii* strain CS03) and compared with the annotated gene model. For RNA-seq analysis, the gene expression level was estimated by counting the sequence reads located in the genome region or gene exon region. Then, DESeq2 was used to carry out gene expression difference analysis, study the functions of differentially expressed genes, and visualize the results of differential expression analysis.

### Potential biomarker virulence assay

The differential metabolites were dissolved in sterile water at 1 mg/mL. Ten microliters of the final solution was injected into the body of *G. mellonella* and dripped onto the body surface of *B. dorsalis* to verify its toxicity. The other treatments were the same as those described above.

### Statistical analysis

The corrected mortality (*M*_*c*_) was calculated using the following formula: *M*_*c*_=(*M*_tre_ *− M*_ctrl_)/(100 − *M*_ctrl_) (39), where *M*_tre_ and *M*_ctrl_ are the mean mortality treatment and control, respectively.

The following logistic function was used in nonlinear fitting for cohort’s mortality: *y* = *A*2 + (*A*1 − *A*2)/(1 + [*x*/*x*0]^*p*), where *y*, *x*, *x*0, *A*1, *A*2, and *p* are the mean dependent variables, independent variables, center, unit value, final value, and power, respectively.

ImageJ software 2 (Wayne Rasband, National Institutes of Health, Bethesda, MD, USA) was used to visualize and measure nematode eggs, body length, body width, etc. One-way ANOVA was used to analyze all obtained data using SPSS software 25.0 (SPSS Inc., Chicago, IL, USA). Origin software 2022 (OriginLab Inc., Massachusetts, USA) was used to perform logistic analysis, nonlinear fitting, multivariate statistical analyses, PCA, mortality mapping, and preparing heatmaps and other graphs.

## Data Availability

The sequencing data set for transcriptome has been submitted to the National Center for Biotechnology Information; the accession number is PRJNA996369.

## SUPPLEMENTAL MATERIAL

Supplemental figures spectrum.01422-23-s0001.docx Fig. S1 and S2.

ASM does not own the copyrights to Supplemental Material that may be linked to, or accessed through, an article. The authors have granted ASM a non-exclusive, world-wide license to publish the Supplemental Material files. Please contact the corresponding author directly for reuse.

## References

[1] Campos-Herrera R , Vicente-Díez I , Galeano M , Chelkha M , Del Mar González-Trujillo M , Puelles M , Labarga D , Pou A , Calvo J , Belda JE . 2021. Intraspecific virulence of entomopathogenic nematodes against the Pests frankliniella occidentalis (Thysanoptera: Thripidae) and Tuta absoluta (Lepidoptera: Gelechiidae). J Nematol 53:e2021-102. doi:10.21307/jofnem-2021-102 PMC8672422 34957410

[2] Terzidis AN , Wilcockson S , Leifert C . 2014. The tomato leaf miner (Tuta absoluta): Conventional pest problem, organic management solutions. Org Agr 4:43–61. doi:10.1007/s13165-014-0064-4

[3] van der Linden CFH , Fatouros NE , Kammenga JE . 2022. The potential of entomopathogenic nematodes to control moth pests of ornamental plantings. Biological Control 165:104815. doi:10.1016/j.biocontrol.2021.104815

[4] Ehlers RU . 2001. Mass production of entomopathogenic nematodes for plant protection. Appl Microbiol Biotechnol 56:623–633. doi:10.1007/s002530100711 11601608

[5] Sicard M , Brugirard-Ricaud K , Pagès S , Lanois A , Boemare NE , Brehélin M , Givaudan A . 2004. Stages of infection during the tripartite interaction between Xenorhabdus nematophila, its nematode vector, and insect hosts. Appl Environ Microbiol 70:6473–6480. doi:10.1128/AEM.70.11.6473-6480.2004 15528508 PMC525208

[6] Koppenhöfer AM , Grewal PS , Fuzy EM . 2007. Differences in penetration routes and establishment rates of four entomopathogenic nematode species into four white grub species. J Invertebr Pathol 94:184–195. doi:10.1016/j.jip.2006.10.005 17156793

[7] Sugar DR , Murfin KE , Chaston JM , Andersen AW , Richards GR , deLéon L , Baum JA , Clinton WP , Forst S , Goldman BS , Krasomil-Osterfeld KC , Slater S , Stock SP , Goodrich-Blair H . 2012. Phenotypic variation and host interactions of Xenorhabdus bovienii SS-2004, the entomopathogenic symbiont of Steinernema jollieti nematodes. Environ Microbiol 14:924–939. doi:10.1111/j.1462-2920.2011.02663.x 22151385 PMC3307839

[8] Kim SK , Flores-Lara Y , Patricia Stock S . 2012. Morphology and ultrastructure of the bacterial receptacle in Steinernema nematodes (Nematoda: Steinernematidae). J Invertebr Pathol 110:366–374. doi:10.1016/j.jip.2012.04.011 22564260

[9] Balcerzak M . 1992. Comparative studies onParasitism caused by Entomogenous nematodes, Steinernema feltiae and Heterorhabditis bacteriophora. Ⅱ.Effects on immune responses of the host insect. Acta Parasitologica Polonica 37:29–35.

[10] Blow F , Douglas AE . 2019. The hemolymph microbiome of insects. J Insect Physiol 115:33–39. doi:10.1016/j.jinsphys.2019.04.002 30953618

[11] Bhatt P , Bhatt K , Huang Y , Lin Z , Chen S . 2020. Esterase is a powerful tool for the biodegradation of pyrethroid insecticides. Chemosphere 244:125507. doi:10.1016/j.chemosphere.2019.125507 31835049

[12] Liu XM , Cao AC , Yan DD , Ouyang CB , Wang QX , Li Y . 2021. Overview of mechanisms and uses of biopesticides. Int J Pest Manag 67:65–72. doi:10.1080/09670874.2019.1664789

[13] Gaugler R , Kaya HK. Entomopathogenic nematodes in biological control. New Jersey, USA:CRC Press. 1990.

[14] Gotz P , Boman A , Boman HG . 1981. Interactions between insect immunity and an insect-pathogenic nematode with symbiotic bacteria. Proc R Soc Lond B 212:333–350. doi:10.1098/rspb.1981.0043

[15] Akhurst RJ , Smigielski AJ , Mari J , Boemare N , Mourant RG . 1992. Restriction analysis of phase variation in Xenorhabdus Spp (Enterobacteriaceae), entomopathogenic bacteria associated with nematodes. Systematic and Applied Microbiology 15:469–473. doi:10.1016/S0723-2020(11)80224-9

[16] Hurlbert RE , Xu JM , Small CL . 1989. Colonial and cellular polymorphism in Xenorhabdus-luminescens . Appl Environ Microbiol 55:1136–1143. doi:10.1128/aem.55.5.1136-1143.1989 16347906 PMC184266

[17] Moureaux N , Karjalainen T , Givaudan A , Bourlioux P , Boemare N . 1995. Biochemical-characterization and agglutinating properties of Xenorhabdus-nematophilus F1 Fimbriae. Appl Environ Microbiol 61:2707–2712. doi:10.1128/aem.61.7.2707-2712.1995 16535079 PMC1388497

[18] Brivio MF , Mastore M . 2018. Nematobacterial complexes and insect hosts: different weapons for the same war. Insects 9:117. doi:10.3390/insects9030117 30208626 PMC6164499

[19] Abebew D , Sayedain FS , Bode E , Bode HB . 2022. Uncovering nematicidal natural products from Xenorhabdus bacteria. J Agric Food Chem 70:498–506. doi:10.1021/acs.jafc.1c05454 34981939 PMC8778618

[20] Tobias NJ , Wolff H , Djahanschiri B , Grundmann F , Kronenwerth M , Shi Y-M , Simonyi S , Grün P , Shapiro-Ilan D , Pidot SJ , Stinear TP , Ebersberger I , Bode HB . 2017. Natural product diversity associated with the nematode symbionts Photorhabdus and Xenorhabdus. Nat Microbiol 2:1676–1685. doi:10.1038/s41564-017-0039-9 28993611

[21] Boemare NE , Akhurst RJ . 1988. Biochemical and physiological characterization of colony form variants in Xenorhabdus spp (Enterobacteriaceae). Microbiology 134:751–761. doi:10.1099/00221287-134-3-751

[22] Smits WK , Kuipers OP , Veening JW . 2006. Phenotypic variation in bacteria: the role of feedback regulation. Nat Rev Microbiol 4:259–271. doi:10.1038/nrmicro1381 16541134

[23] Shi YM , Bode HB . 2018. Chemical language and warfare of bacterial natural products in bacteria-nematode-insect interactions. Nat Prod Rep 35:309–335. doi:10.1039/c7np00054e 29359226

[24] Morgan JAW , Sergeant M , Ellis D , Ousley M , Jarrett P . 2001. Sequence analysis of insecticidal genes from Xenorhabdus nematophilus PMFI296 . Appl Environ Microbiol 67:2062–2069. doi:10.1128/AEM.67.5.2062-2069.2001 11319082 PMC92837

[25] Kinkar OU , Prashar A , Kumar A , Hadapad AB , Hire RS , Makde RD . 2022. Txp40, an Insecticidal toxin protein from Xenorhabdus nematophila: purification, toxicity assessment and biophysical characterization. Toxicon 218:40–46. doi:10.1016/j.toxicon.2022.09.003 36096207

[26] Cowles KN , Goodrich-Blair H . 2005. Expression and activity of a Xenorhabdus nematophila haemolysin required for full virulence towards manduca sexta insects. Cell Microbiol 7:209–219. doi:10.1111/j.1462-5822.2004.00448.x 15659065

[27] McInerney BV , Taylor WC , Lacey MJ , Akhurst RJ , Gregson RP . 1991. Biologically-active metabolites from Xenorhabdus spp.2. benzopyran-1-one derivatives with gastroprotective activity. J. Nat. Prod 54:785–795. doi:10.1021/np50075a006 1955881

[28] Huot L , George S , Girard P-A , Severac D , Nègre N , Duvic B . 2019. Spodoptera frugiperda transcriptional response to infestation by Steinernema carpocapsae. Sci Rep 9:12879. doi:10.1038/s41598-019-49410-8 31501491 PMC6733877

[29] Gaugler R . 2002. Entomopathogenic Nematology. In Bacterial metabolites. CABI Publishing, UK. doi:10.1079/9780851995670.0000

[30] Zhou Q , Grundmann F , Kaiser M , Schiell M , Gaudriault S , Batzer A , Kurz M , Bode HB . 2013. Structure and biosynthesis of xenoamicins from entomopathogenic Xenorhabdus. Chemistry 19:16772–16779. doi:10.1002/chem.201302481 24203528

[31] Kronenwerth M , Bozhüyük KAJ , Kahnt AS , Steinhilber D , Gaudriault S , Kaiser M , Bode HB . 2014. Characterisation of taxlllaids a-g. natural products from Xenorhabdus indica *.* Chemistry 20:17478–17487. doi:10.1002/chem.201403979 25351611

[32] Lang G , Kalvelage T , Peters A , Wiese J , Imhoff JF . 2008. Linear and cyclic peptides from the entomopathogenic bacterium Xenorhabdus nematophilus *.* J Nat Prod 71:1074–1077. doi:10.1021/np800053n 18491867

[33] Nollmann FI , Dowling A , Kaiser M , Deckmann K , Grösch S , Ffrench-Constant R , Bode HB . 2012. Synthesis of szentiamide, a depsipeptide from entomopathogenic Xenorhabdus szentirmaii with activity against Plasmodium falciparum. Beilstein J Org Chem 8:528–533. doi:10.3762/bjoc.8.60 22563351 PMC3343279

[34] Grundmann F , Kaiser M , Kurz M , Schiell M , Batzer A , Bode HB . 2013. Structure determination of the bioactive depsipeptide xenobactin from Xenorhabdus sp. RSC Adv 3:22072. doi:10.1039/c3ra44721a

[35] McInerney BV , Gregson RP , Lacey MJ , Akhurst RJ , Lyons GR , Rhodes SH , Smith DR , Engelhardt LM , White AH . 1991. biologically active metabolites from Xenorhabdus spp. 1. Dithiolopyrrolone derivatives with antibiotic activity. J Nat Prod 54:774–784. doi:10.1021/np50075a005 1955880

[36] Fuchs SW , Grundmann F , Kurz M , Kaiser M , Bode HB . 2014. Fabclavines: bioactive peptide–polyketide-polyamino hybrids from Xenorhabdus. Chembiochem 15:512–516. doi:10.1002/cbic.201300802 24532262

[37] Brachmann AO , Reimer D , Lorenzen W , Augusto Alonso E , Kopp Y , Piel J , Bode HB . 2012. Reciprocal cross talk between fatty acid and antibiotic biosynthesis in a nematode symbiont. Angew Chem Int Ed Engl 51:12086–12089. doi:10.1002/anie.201205384 23097192

[38] Reimer D , Nollmann FI , Schultz K , Kaiser M , Bode HB . 2014. Xenortide biosynthesis by entomopathogenic Xenorhabdus nematophila. J Nat Prod 77:1976–1980. doi:10.1021/np500390b 25080196

[39] Bi YH , Gao CZ , Yu ZG . 2018. Rhabdopeptides from Xenorhabdus Budapestensis SN84 and their nematicidal activities against Meloidogyne incognita *.* J Agric Food Chem 66:3833–3839. doi:10.1021/acs.jafc.8b00253 29597344

[40] Fuchs SW , Sachs CC , Kegler C , Nollmann FI , Karas M , Bode HB . 2012. Neutral loss fragmentation pattern based screening for arginine-rich natural products in Xenorhabdus and Photorhabdus. Anal Chem 84:6948–6955. doi:10.1021/ac300372p 22873683

[41] Fuchs SW , Proschak A , Jaskolla TW , Karas M , Bode HB . 2011. Structure elucidation and biosynthesis of lysine-rich cyclic peptides in Xenorhabdus nematophila *.* Org Biomol Chem 9:3130–3132. doi:10.1039/c1ob05097d 21423922

[42] Houard J , Aumelas A , Noël T , Pages S , Givaudan A , Fitton-Ouhabi V , Villain-Guillot P , Gualtieri M . 2013. Cabanillasin, a new antifungal metabolite, produced by entomopathogenic Xenorhabdus cabanillasii Jm26. J Antibiot 66:617–620. doi:10.1038/ja.2013.58 23756685

[43] Morales-Soto N , Gaudriault S , Ogier JC , Thappeta KRV , Forst S . 2012. Comparative analysis of P2-type remnant prophage loci in Xenorhabdus bovienii and Xenorhabdus nematophila required for Xenorhabdicin production. FEMS Microbiol Lett 333:69–76. doi:10.1111/j.1574-6968.2012.02600.x 22612724

[44] Singh J , Banerjee N . 2008. Transcriptional analysis and functional characterization of a gene pair Encoding iron-regulated Xenocin and immunity proteins of Xenorhabdus nematophila *.* J Bacteriol 190:3877–3885. doi:10.1128/JB.00209-08 18375563 PMC2395030

[45] Li JX , Chen GH , Webster JM . 1997. Nematophin, a novel antimicrobial substance produced by Xenorhabdus nematophilus (Enterobactereaceae). Can J Microbiol 43:770–773. doi:10.1139/m97-110 9304787

[46] Crawford JM , Portmann C , Zhang X , Roeffaers MBJ , Clardy J . 2012. Small molecule perimeter defense in entomopathogenic bacteria. Proc Natl Acad Sci U S A 109:10821–10826. doi:10.1073/pnas.1201160109 22711807 PMC3390839

[47] Spogliarich R , Farnetti E , Kašpar J , Graziani M , Cesarotti E . 1989. Selective hydrogenation of benzylideneacetone catalyzed by iridium diphosphine complexes. J Mol Catal 50:19–29. doi:10.1016/0304-5102(89)80106-3

[48] Brachmann AO , Forst S , Furgani GM , Fodor A , Bode HB . 2006. Xenofuranones a and b: phenylpyruvate dimers from Xenorhabdus szentirmaii *.* J Nat Prod 69:1830–1832. doi:10.1021/np060409n 17190473

[49] Oliva B , O’Neill A , Wilson JM , O’Hanlon PJ , Chopra I . 2001. Antimicrobial properties and mode of action of the pyrrothine holomycin. Antimicrob Agents Chemother 45:532–539. doi:10.1128/AAC.45.2.532-539.2001 11158751 PMC90323

[50] Bode E , He Y , Vo TD , Schultz R , Kaiser M , Bode HB . 2017. Biosynthesis and function of simple amides in Xenorhabdus doucetiae. Environ Microbiol 19:4564–4575. doi:10.1111/1462-2920.13919 28892274

[51] Vilcinskas A . 2013. Yellow biotechnology I, p 123–155. In Vilcinskas A (ed), Identification and Bioanalysis of natural products from insect Symbionts and pathogens. Springer International Publishing AG, Berlin, Heidelberg. doi:10.1007/978-3-642-39863-6 23657492

[52] Dreyer J , Malan AP , Dicks LMT . 2018. Bacteria of the genus Xenorhabdus, a novel source of bioactive compounds. Front Microbiol 9:3177. doi:10.3389/fmicb.2018.03177 30619229 PMC6305712

[53] Troost T , Hitchcock MJ , Katz E . 1980. Distinct kynureninase and hydroxykynureninase enzymes in an actinomycin-producing strain of Streptomyces-parvulus *.* Biochim Biophys Acta 612:97–106. doi:10.1016/0005-2744(80)90282-x 6153909

[54] Olsson A , Diaz T , Aguilar-Santelises M , Osterborg A , Celsing F , Jondal M , Osorio LM . 2001. Sensitization to TRAIL-induced apoptosis and modulation of FLICE-inhibitory protein in b chronic lymphocytic leukemia by Actinomycin D. Leukemia 15:1868–1877. doi:10.1038/sj.leu.2402287 11753607

[55] Zhang Y , Wang F , Zhao ZH . 2022. Reveals that entomopathogenic nematodes mediate tryptophan metabolites that kill host insects. Front Microbiol 13:1042145. doi:10.3389/fmicb.2022.1042145 36439848 PMC9686292

[56] Eliáš S , Hurychová J , Toubarro D , Frias J , Kunc M , Dobeš P , Simões N , Hyršl P . 2020. Bioactive excreted/secreted products of entomopathogenic nematodeheterorhabditis bacteriophorainhibit the phenoloxidase activity during the infection. Insects 11:353. doi:10.3390/insects11060353 32516962 PMC7349556

[57] Salem HM , Hussein MA , Hafez Se , Hussein MA , Sayed RM . 2020. Hemocytic studies on the synergistic effect of the entomopathogenic Nematode species, Steinernema Carpocapsae and gamma radiation on the greater wax moth, Galleria Mellonella (L.) larvae. Egypt J Biol Pest Control 30:48. doi:10.1186/s41938-020-00254-9

[58] Ribeiro C , Vignes M , Brehélin M . 2003. Xenorhabdus nematophila (enterobacteriacea) secretes a cation-selective calcium-independent porin which causes vacuolation of the rough endoplasmic reticulum and cell lysis. J Biol Chem 278:3030–3039. doi:10.1074/jbc.M210353200 12441337

[59] Péchy-Tarr M , Bruck DJ , Maurhofer M , Fischer E , Vogne C , Henkels MD , Donahue KM , Grunder J , Loper JE , Keel C . 2008. Molecular analysis of a novel gene cluster encoding an insect toxin in plant-associated strains of Pseudomonas fluorescens *.* Environ Microbiol 10:2368–2386. doi:10.1111/j.1462-2920.2008.01662.x 18484997

[60] Jing Y , Toubarro D , Hao Y , Simões N . 2010. Cloning, characterisation and heterologous expression of an astacin metalloprotease, Sc-AST, from the entomoparasitic nematode Steinernema carpocapsae *.* Mol Biochem Parasitol 174:101–108. doi:10.1016/j.molbiopara.2010.07.004 20670659

[61] Nedeva C , Menassa J , Duan M , Liu C , Doerflinger M , Kueh AJ , Herold MJ , Fonseka P , Phan TK , Faou P , Rajapaksha H , Chen W , Hulett MD , Puthalakath H . 2020. Treml4 receptor regulates inflammation and innate immune cell death during polymicrobial sepsis. Nat Immunol 21:1585–1596. doi:10.1038/s41590-020-0789-z 33020659

[62] Bitzer J , Gesheva V , Zeeck A . 2006. Actinomycins with altered threonine units in the beta-peptidolactone. J Nat Prod 69:1153–1157. doi:10.1021/np060063g 16933866

[63] Paramanathan T , Vladescu I , McCauley MJ , Rouzina I , Williams MC . 2012. Force spectroscopy reveals the DNA structural dynamics that govern the slow binding of actinomycin d. Nucleic Acids Res 40:4925–4932. doi:10.1093/nar/gks069 22328730 PMC3367174

[64] Gaertner FH , Shetty AS . 1977. Kynureninase-type enzymes and evolution of aerobic tryptophan to nicotinamide adenine-dinucleotide pathway. Biochim Biophys Acta 482:453–460. doi:10.1016/0005-2744(77)90259-5 195620

[65] Davis RA , Miyake JH , Hui TY , Spann NJ . 2002. Regulation of cholesterol-7α-hydroxylase: barely missing a SHP. J Lipid Res 43:533–543. 11907135

[66] St-Pierre MV , Kullak-Ublick GA , Hagenbuch B , Meier PJ . 2001. Transport of bile acids in hepatic and non-hepatic tissues. J Exp Biol 204:1673–1686. doi:10.1242/jeb.204.10.1673 11316487

[67] Li FL , Liu HZ , Xi XD , Yu ZG . 2018. Secondary metabolites of Xenorhabdus Nematophila SN313 and their inhibitory activities against plant pathogenic fungi. Chin J Pestic Sci 20:163–168.

[68] Martens EC , Heungens K , Goodrich-Blair H . 2003. Early colonization events in the mutualistic association between Steinernema carpocapsae nematodes and Xenorhabdus nematophila bacteria. J Bacteriol 185:3147–3154. doi:10.1128/JB.185.10.3147-3154.2003 12730175 PMC154081

[69] Navon A , Ascher KRS . 2000. Bioassays of Entomopathogenic Microbes and nematodes. In Bioassays for Entomopathogenic nematodes. CABI Publishing, UK. doi:10.1079/9780851994222.0000

[70] Gu XY , Zhao Y , Su Y , Wu JJ , Wang ZY , Hu JT , Liu LJ , Zhao ZH , Hoffmann AA , Chen B , Li ZH . 2019. transcriptional and functional analysis of heat hardening in two invasive fruit fly species, Bactrocera dorsalis and Bactrocera correcta. Evol Appl 12:1147–1163. 31293628 10.1111/eva.12793 PMC6597872

[71] Akhurst RJ . 1980. Morphological and functional dimorphism in Xenorhabdus spp bacteria symbiotically associated with the insect pathogenic nematodes Neoaplectana and heterorhabditis. Microbiology 121:303–309. doi:10.1099/00221287-121-2-303

[72] McMullen JG , McQuade R , Ogier J-C , Pagès S , Gaudriault S , Patricia Stock S . 2017. Variable virulence phenotype of Xenorhabdus bovienii (γ-proteobacteria: enterobacteriaceae) in the absence of their vector hosts. Microbiology (Reading) 163:510–522. doi:10.1099/mic.0.000449 28430102 PMC7008216

[73] Cao J , Li M , Chen J , Liu P , Li Z . 2016. Effects of meja on arabidopsis Metabolome under endogenous JA deficiency. Sci Rep 6:37674. doi:10.1038/srep37674 27883040 PMC5121592

