# Supplementary material for: Discovery of a new highly pathogenic toxin involved in insect sepsis
Source: Microbiol Spectr. 2023 Oct 3;11(6):e01422-23. doi: 10.1128/spectrum.01422-23 (PMC10715044; doi:10.1128/spectrum.01422-23)
Supplement: Supplemental figures — Fig. S1 and S2. [file spectrum.01422-23-s0001.docx]

**Figure S1.** Effect of *S. feltiae* exposure of *B. dorsalis.* (A) Temperature-dependent effects of *S. feltiae* exposure on the mortality of *B. dorsalis*. (B) Effect of *S. feltiae* exposure on the mortality of *B. dorsalis* at different doses.





**Figure S2.** Toxicity of potential biomarkers in *B. dorsalis*. In total, 40 substances from metabolome analysis were tested in the survival analysis.
